# “Make Stories That Will Always Be There”: Eagle Books’ Appeal, Sustainability, and Contributions to Public Health, 2006–2022

**DOI:** 10.5888/pcd20.220315

**Published:** 2023-04-13

**Authors:** Dawn Satterfield, Lemyra DeBruyn, Teresa Lofton, Carolee Dodge Francis, Virginie Zoumenou, Lorelei DeCora, Chelsea Wesner

**Affiliations:** 1 Native Diabetes Wellness Program, Division of Diabetes Translation, Centers for Disease Control and Prevention, Atlanta, Georgia; 2 Westat, Inc, Atlanta, Georgia; 3 School of Human Ecology, University of Wisconsin-Madison; 4 University of Maryland Eastern Shore Extension, Princess Anne, Maryland; 5 Winnebago Tribe of Nebraska, Winnebago, Nebraska; 6 Center for American Indian and Alaska Native Health, University of Colorado Anschutz Medical Campus, Aurora, Colorado

## Abstract

**Purpose and Objectives:**

We aimed to determine why the Eagle Books, an illustrated series for American Indian and Alaska Native (AIAN) children to address type 2 diabetes, remain viable long after their release. We sought to answer 2 questions: Why did the books maintain popularity? What factors have sustained them?

**Intervention Approach:**

Type 2 diabetes burgeoned in the US after World War II, compounding a long legacy of injustices for AIAN peoples. By the 1980s, their rates soared above those of White people. Concerned for future generations, Tribal Leaders suggested that the Centers for Disease Control and Prevention and Indian Health Service use traditional storytelling to teach children about staying healthy. Public health interventions are most effective when culture and history are integrated into health education, particularly stories to address a relatively new disease for AIAN peoples.

**Evaluation Methods:**

From 2008 through 2013, we conducted a case study among 8 tribal communities to evaluate the uptake of the Eagle Books across Indian Country. To understand the Eagle Books’ sustained appeal, in 2022 we reanalyzed the original case study themes and analyzed for the first time themes that emerged from evaluation results in the Eagle Books’ program literature. These were programs that had independently evaluated their use of the Eagle Books and published their findings.

**Results:**

Outcomes demonstrated continuous application of the Eagle Books in diverse community interventions, influencing children's healthy choices. Community implementers described sustainability components, such as the books’ versatility, flexibility of use, and availability online and in print.

**Implications for Public Health:**

Historical, social, economic, and environmental health determinants intersect with biological and behavioral factors to weave a complex web of causation for type 2 diabetes, beginning early in life. Compelling, colorful stories reflecting traditional wisdom and respect for Western and Indigenous science — through the eyes of a wise eagle, a clever rabbit, a tricky coyote, and kids in T-shirts and sneakers — can positively influence community health.

**Summary**

**What is already known on this topic?**

Public health interventions are most effective when culture and history are integrated into health education programs. Memorable stories for children can foster learning about preventing chronic diseases, including type 2 diabetes.

**What is added by this report?**

Sustainability factors include versatility of use, continued availability, culturally relevant messages, compelling illustrations, and cultural identity. Children who “look like today’s kids” with connections to their elders’ traditional knowledge have propelled the Eagle Books’ appeal and longevity for 16 years.

**What are the implications for public health practice?**

Well told, colorful stories based on Indigenous traditional wisdom and honoring the time-tested skill of storytelling can affect children’s healthy choices and, consequently, community health.

## Introduction

Children and adolescents in the US today face a greater risk than previous generations for type 2 diabetes and shortened lifespans, an unprecedented reversal in health (1). Case reports of American Indian and Alaska Native (AIAN) adolescents in the US and Canada with type 2 diabetes surfaced in the 1970s and 1980s, startling medical practitioners who had long considered it an adult disease. By the mid-90s, the epidemic of type 2 diabetes, characterized by insulin resistance and propelled by obesity, had affected children and adolescents in all US populations (2). From 1996 to 2004, type 2 diabetes prevalence among AIAN adolescents aged 15 to 19 years increased by 68% (3). In 2019, AIAN and African American children and adolescents aged 19 or younger had the highest type 2 diabetes rates compared with peers in other US populations (4), placing them at risk for complications such as chronic kidney disease while they are still young (5).

Collective factors, termed social determinants of health (SDOH), can predict physical and mental health outcomes. Socioeconomic status, including economic, educational, and occupational status, is strongly associated with diabetes risks and outcomes (6). For example, obesity prevalence was 18.9% among children and adolescents in the US aged 2 to 19 years in the lowest income group, 19.9% in the middle-income group, and 10.9% among those in the highest income group (7). Connectedness with “place,” which for many AIAN and other peoples encompasses loss of homeland and community (8) also impacts health.

Trauma and chaotic conditions in childhood trigger physiologic stress, leading to neurologic regulatory responses that alter the brain’s pathways (9). Adverse childhood experiences (eg, witnessing violence, personally experiencing abuse or neglect) (10) correlate with obesity and type 2 diabetes across populations (11), including AIAN populations (12). Poverty contributes to conditions that can perpetuate adverse childhood experiences (eg, crowded housing, stress, and food insecurity). In 2020, two racial groups had poverty rates more than 10 percentage points higher than the national rate of 14.3%: AIAN (27.0%) and Black or African American (25.8%) (13). From 2000 to 2010, 25% of AIAN families were consistently food insecure, twice that of White families (14).

Stressors associated with colonization (eg, trauma, loss of lands, relocation to reservations, food insecurity, poverty), compounded across centuries, are linked to trends in obesity and type 2 diabetes in recent decades. As defined in 1998 by Brave Heart and DeBruyn, historical trauma is the collective, complex trauma inflicted on a group of people with a specific group identity or affiliation (eg, ethnicity, nationality, religious affiliation) (15). For generations of AIAN children, harsh conditions in boarding schools also contributed (16). “They taught us to be stingy,” said an elder removed from her home as a young child to attend boarding school. Competition for food to avoid hunger countered her cultural values of generosity and sharing (17).

**Figure 1:**
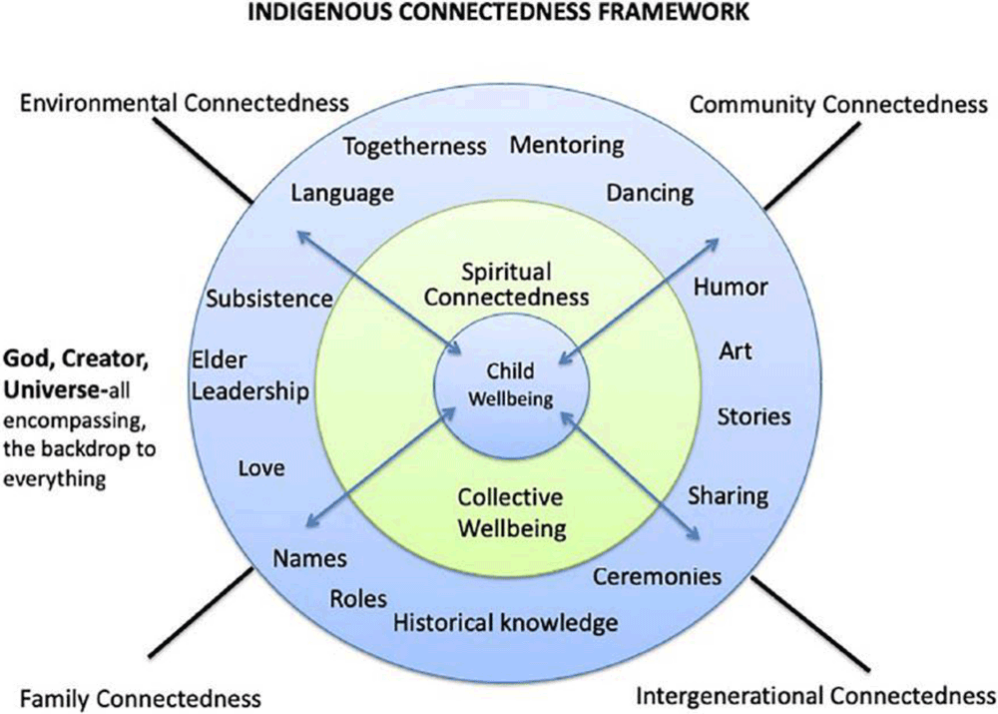
Indigenous connectedness framework for child well-being created by Ullrich (18). Reprinted with permission from the author.

Indigenous peoples’ survival and well-being has been supported by connectedness, the interrelated welfare of everyone and everything (18). Protective factors such as safe, stable, and nurturing relationships (10) can serve as buffers that mediate stressful and traumatic life events (19,20). Strengths-based health promotion efforts, including type 2 diabetes prevention programming, leverage protective factors to foster connectedness across environments and support the health and relational well-being of AIAN children and adolescents (20,21). Indigenous scholars note that historical and protective factors influence all levels of socioecological models, increasing a sense of belonging, self-esteem, self-efficacy, and health knowledge (1,18,21–24). For example, an Indigenous connectedness framework created by Ullrich (Figure 1) centers on child well-being in the context of intergenerational, environmental, family, and community connectedness, encompassed by spiritual and cultural connectedness (18). Stories, dance, music, and ceremony are common expressions of connectedness across Indigenous cultures.

Concerned for their people and future generations, tribal leaders and allies testified about the disproportionate prevalence of diabetes before Congress, which passed the Balanced Budget Act of 1997 (25). Under this legislation, lawmakers established the Special Diabetes Program for Indians in 1998, administered by the Indian Health Service (IHS). The Tribal Leaders Diabetes Committee (TLDC) guided tribally driven, culturally grounded programs designed to advance diabetes care and prevention (26). The Special Diabetes Program for Indians, with more than 300 programs nationwide, continues to demonstrate substantial improvements in health outcomes for AIAN people (27,28). The incidence of type 2 diabetes among AIAN adults decreased 5.2% from 15.4% in 2013 to 14.6% in 2017 (29). Overweight and obesity rates among AIAN children and adolescents aged 2 to 18 years, although high compared with their peers in other racial and ethnic groups, appear to have stabilized in recent years (30).

The IHS provided funds to the Centers for Disease Control and Prevention (CDC) through an interagency agreement, leading to the formation of CDC’s Division of Diabetes Translation Native Diabetes Wellness Program (NDWP) in 2004. The NDWP established principles of practice to inform the program’s work in Indian Country (Box).

**Box. Native Diabetes Wellness Program Principles of Practice**

**Mission**

The mission of the Native Diabetes Wellness Program is to work with a growing circle of partners to address the health inequities so starkly revealed by the number of people with diabetes in Indian Country. With social justice and respect for Indigenous and Western science as grounding principles, we strive to support community efforts to promote health and prevent type 2 diabetes.

**Vision**

Indian Country free of the devastation of diabetes.

**Goals**

1. Support sustainable, evaluable ecological approaches to promote Indigenous knowledge about physical, mental, spiritual, and emotional health, including foods, physical activity, and social support.
2. Share stories that promote health in traditional ways, to be remembered, retold, and talked about in homes, schools, and communities.

**Principles of Practice**

1. Listen.
2. Recognize tribal sovereignty and respect the diversity of tribes.
3. Consult tribal leadership and tribal members.
4. Honor federal responsibility to tribal nations.
5. Respect and incorporate Indigenous science.
6. Share a vision of hope.
7. Honor storytelling and the power of stories.
8. Establish direct relationships with tribal nations.
9. Respect the power of words — keep our word.
10. Seek reciprocity and balance.
11. Be grateful for our work.
12. Reflect critically.
13. Practice cultural humility.

The Diabetes Prevention Program (DPP), a landmark clinical trial to determine if treatment with lifestyle changes or medication can help prevent type 2 diabetes, was published in 2002 (31). AIAN adults living in the Southwest joined other volunteers with prediabetes for the study, supported by the National Institutes of Health, CDC, and IHS, confirming that type 2 diabetes can often be prevented with intensive lifestyle interventions (31). The Special Diabetes Program for Indians successfully replicated the DPP in tribal-based reservation and urban communities (32).

**Figure 2:**
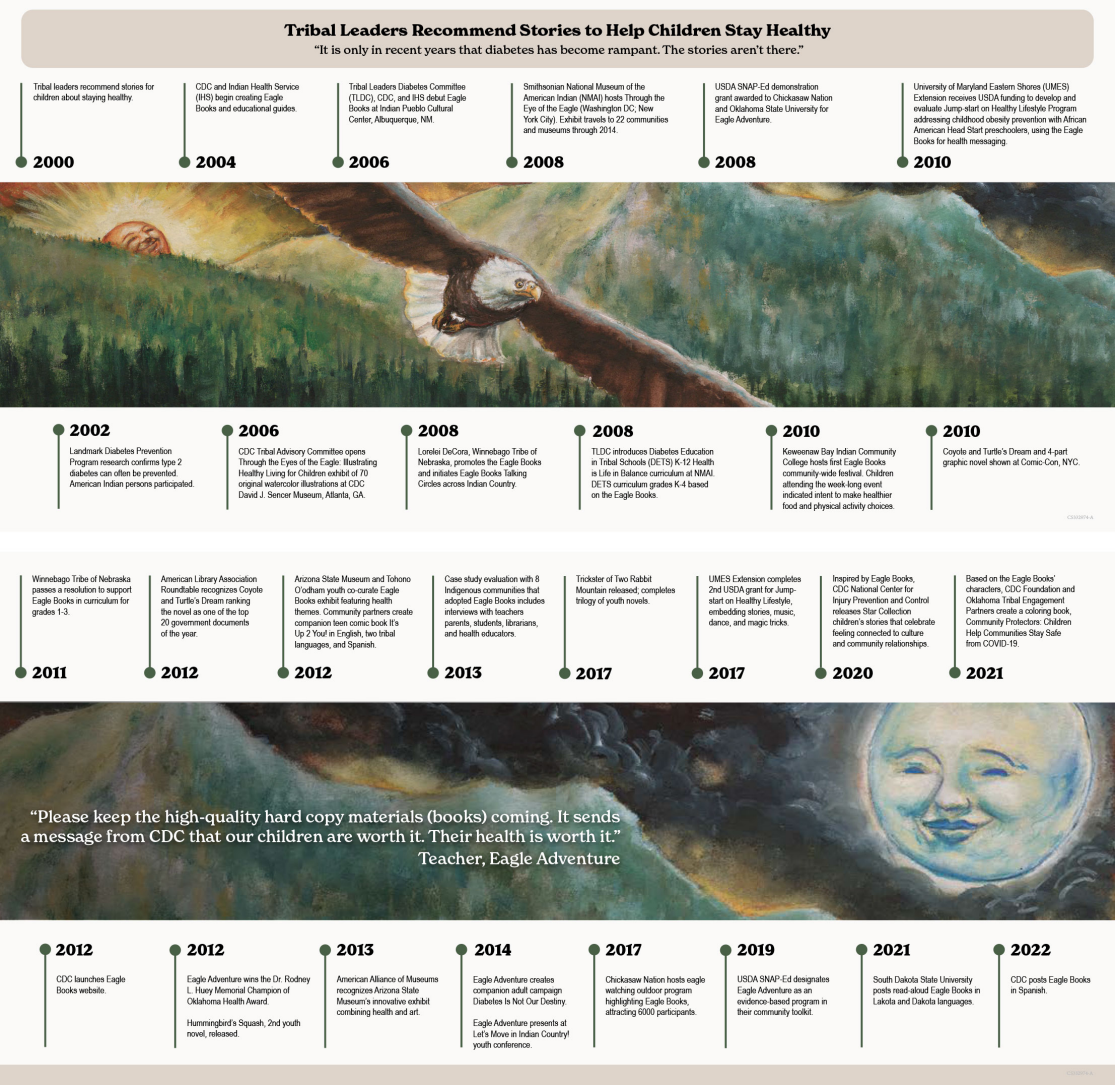
A timeline of the development and implementation of Eagle Books, 2006–2022. [A text version of this figure is available.] Abbreviation: USDA SNAP-Ed, US Department of Agriculture Supplemental Nutrition Assistance Program–Education.

In 2000 and 2001, IHS, CDC, and TLDC held 8 listening sessions. More than 421 representatives from 171 tribes offered guidance on community-based approaches for diabetes prevention and care. A recurring theme was respect for traditional knowledge about protecting people's health and appreciating the diversity of tribes. “Look to the culture. Our cultures are the source of health,” one representative said. Related to this was a deep concern for the health of children, who are considered sacred (33) in many AIAN cultures. “We need stories . . . it’s just the last decades where [diabetes] has run rampant. The stories aren’t there,” one representative explained. Another added, “Make it [a story] something that is there all the time” (CDC, unpublished report, *Formative Research to Obtain Tribal Input on the National Diabetes Prevention Center*. Westat, Inc, for CDC Division of Diabetes Translation, through CDC Health Communication Evaluation Services; 2000). Several representatives suggested a story about an eagle, told by Georgia Perez, a community health representative for Nambe Pueblo, incorporated into the Strong in Body and Spirit program. Told at the beginning of each session, the story facilitated open discussions. “It was as though walls of guilt, fear, anger, and denial came down, and people had new hope” (34). From 2002 through 2006, *Through the Eyes of the Eagle, Knees Lifted High, Plate Full of Color*, and *Tricky Treats* were written by Georgia Perez and illustrated by Patrick Rolo and Lisa A. Fifield. CDC supported the development of the series through a contract with Westat, Inc. The books feature children in sneakers and T-shirts, a wise eagle, a clever rabbit, and a wily coyote. The books were launched in 2006 at the Indian Pueblo Cultural Center in Albuquerque, New Mexico, and news media outlets covered the event (eg, *Indian Country Today*, *Green Bay Gazette*, *USA Today*). The development and implementation of Eagle Books programs and applications has spanned 2006 through 2022 (Figure 2).

Responding to TLDC guidance, NIH concurrently established cooperative agreements with 8 tribal colleges and universities, and interagency agreements with CDC and IHS to create the K–12 Diabetes Education in Tribal Schools (DETS) curriculum (35,36). The K–4 curriculum included the Eagle Books. NDWP (D.S., L.D.B.) provided scientific review throughout the curriculum development.

In 2010, NDWP created novels for adolescents based on the original series (Figure 2). The children now aged 12 years and the animal characters returned with an expanded cast, including an elderly box turtle and a multicultural trickster rabbit. Written by Terry Lofton and illustrated by Patrick Rolo, these novels broadened the dialogue about type 2 diabetes prevention. Although not included in our evaluation, these books are part of Eagle Books’ continuity.

## Purpose and Objectives

Our study aimed to determine why the Eagle Books, an illustrated series created for AIAN children to address type 2 diabetes, remain viable 16 years after their launch. We sought to answer 2 questions: Why have Eagle Books maintained popularity? What factors sustained them?

The objectives of the Eagle Books were to 1) address the need for diabetes education for AIAN children, 2) create compelling, relevant stories about staying healthy and preventing type 2 diabetes, 3) promote traditional ways of knowing about healthy foods and physical activity through storytelling, and 4) portray vivid images and memorable characters to inspire healthy choices.

## Intervention Approach

We used 2 evaluation approaches. First, we reviewed the qualitative case study of the adoption of the Eagle Books for children in 8 diverse AIAN communities. Second, we performed an implementation evaluation to determine why and how the Eagle Books have remained popular in Indian Country and elsewhere.

### Initial case study in 8 American Indian and Alaska Native communities

From 2008 through 2013, NDWP contracted with Westat to conduct a qualitative case study among AIAN communities to determine their uptake of the Eagle Books since 2006 (Teresa Lofton, PhD, et al, unpublished report, 2013. *Uptake of the Eagle Books in Selected American Indian and Alaska Native Communities: Internal Report*. Supported by the Native Diabetes Wellness Program, Division of Diabetes Translation, CDC. Task order contract no. 200-2007-20015). We chose sites to learn common patterns of use and unique local applications. Selection criteria included variation by culture, geographic region, population size, and whether communities had been exposed to federally funded promotion of the Eagle Books (DETS curriculum, Eagle Book campaign fairs and exhibits, diabetes talking circles) or had independently ordered at least 1,000 books. We wanted to understand how federal promotion influenced uptake and to identify contextual factors that affected the books’ use with or without support. Ultimately, we selected 8 locations — 4 locations had federal support and 4 locations were independent.

Westat assembled a team (T.L., lead evaluator) to conduct evaluation activities on site (C.D.F., L.D.C., and D.S., L.D.B., observers). Community-based and tribally driven participatory research (37) framed our approach, as these tribal partners had determined the most culturally appropriate uptake of the books in their communities.

### Implementation evaluation framework

An implementation evaluation provided the best approach for the present study. We wanted to determine if the intervention, uptake of the Eagle Books, had been implemented in diverse settings and programs to address type 2 diabetes and had influenced children’s healthy food choices. We wanted to learn whether use of the Eagle Books had accomplished the original goals for their use, and if additional findings related to those goals. We were particularly interested in what happened after the Eagle Books case study was completed and what led to the books’ continued viability. Implementation evaluation covered these areas of inquiry to help us understand how sustainability occurred over 16 years.

Funding support for communications about Eagle Books (eg, newsletters and conferences) concluded in 2016. Programs continued to use the books in schools, communities, and culture camps (38). A small number of those programs conducted formal evaluations with similar (qualitative) or other (quantitative or both quantitative and qualitative) methods. Implementation evaluation can use both quantitative and qualitative measures to answer descriptive (who, what, where, when) questions and qualitative measures to explain how and why. Implementation evaluation helped frame the examination of what factors led to continued use of the Eagle Books and if themes from the first assessment still held.

## Evaluation Methods

Methods for the present study are based on the findings of the case study, which we reviewed and reanalyzed to answer our 2 research questions (Table 1). The review was sufficient to address the first question about popularity. To answer the second question about sustainability, 4 raters (T.L., D.S., C.W., L.D.B.) independently reanalyzed these data to identify the most important themes.

**Table 1:** Case Study Methods for Evaluation of Eagle Books, 2008–2013

Phase	Task
**Preparation**	
Developing study protocols and instruments	We developed semistructured interview and focus group schedules, informed consent forms, and site visit recruitment and scheduling sheets for each type of participant.
	We designed structured and open-ended questions in semistructured interview guides to interview health workers (nurses, public health and Indian Health Service staff, diabetes educators, and fitness specialists), school administrators and counselors, and teachers (grades kindergarten through 4).
Purpose	These instruments would assess awareness of Eagle Books in the community, Eagle Books activities in health programs and schools, ease of use and comprehension by children, and how Eagle Books compared with other diabetes prevention materials in appeal and messages. Instruments included questions about barriers for use and ways the Centers for Disease Control and Prevention could improve support of Eagle Books.
Focus groups	Focus group guides were designed for parents. Children (grades kindergarten–4) accompanying their parents would be interviewed with a short discussion about the Eagle Books (what Mr. Eagle wants you to do and why), followed by hands-on activities.
	Focus group questions centered on use and influence of Eagle Books in the home: reading books with children, using messages to encourage children’s healthy behaviors, adoption of healthier food choices and increased physical activity, what they have learned about diabetes prevention, challenges encountered, and general questions about appeal of Eagle Books.
Approvals	Office of Management and Budget (OMB) approved all data collection instruments (OMB no. 0920–0798). All instruments and protocols were approved by the institutional review boards of the Centers for Disease Control and Prevention, Westat, and tribes with institutional review board committees.
Preparing to work with tribal communities	Because some staff did not have experience in Indian Country, Westat held meetings to discuss working with tribal communities. We asked tribal communities to collaborate with us in conducting the site visits. To ensure our interactions were aligned with the principles of participatory research and NDWP’s principles of practice, we introduced inexperienced team members to Native communities’ history, cultures, economics, and proper etiquette. With appreciation for the values of respect, reciprocity, and cultural humility, we adopted qualities of “talking circles” to conduct focus groups and enhance the semistructured interviews: The moderator and participants listened attentively and respectfully to everyone, and speakers were not hurried. Focus groups and interviews were to be held in comfortable, culturally familiar settings, for people to feel safe to talk about family struggles with diabetes and how they want a better life for their children.
Site selection	We selected sites by cultural and geographic diversity. Four sites had federal support from NDWP to promote Eagle Books, and 4 sites had no federal support and had ordered Eagle Books independently.
Recruitment and scheduling	NDWP and project consultants identified local health department staff and diabetes educators to recruit participants. Westat made introductory telephone calls and sent emails to each contact, with follow-up to identify numbers and kinds of participants, and scheduling or confirming visit dates and times. When asked, Westat directly recruited participants.
**Data collection**	
Methods used (39)	We used qualitative data collection methods: in-depth, semistructured interviews, focus groups, collection of locally developed Eagle Books–related materials, and observational tours.
Participants	Representatives from health departments, schools, colleges, museums, libraries, Native organizations, and cultural programs, in addition to parents/caregivers and children and adolescents, participated in data collection.
Participatory approach	Participants and researchers engaged in conversational interaction with each other. Participants steered the agenda content by asking questions, making recommendations, and expressing opinions.
	This participatory approach embraced the traditions of oral Native communication that encourages respect and equitable co-creation of knowledge.
Value of multiple methods	Multiple cases, data collection methods, and sources provided in-depth data necessary to understand appeal of Eagle Books, initial uptake and continued use, kinds of uptake and their effects, and factors that influenced site-specific use. Interviews and observational tours took place in the natural environment of participants’ own community.
Site visits	Site visits were conducted from October 2011 through June 2012.
	The evaluation team consisted of 3 or 4 researchers: at least 2 Westat staff members (evaluation lead [T.L.], 1 other) and 1 project consultant (C.D.F. or L.D.C.). These researchers conducted focus groups and semistructured interviews.
	NDWP codirectors (D.S. or L.D.B.) attended as observers only.
	The evaluation team interviewed 186 participants; 3 participants in Alaska were interviewed by telephone.
Process	At the beginning of individual, small group, and focus group interviews, participants received a $70 gift card.
	Focus groups began with a blessing by an elder followed by a box lunch. Parents signed consent forms and child permission forms before children went to a separate room for their interview.
	Three members of the team conducted focus groups: a moderator, a note taker to augment audio recording, and a team member to entertain the children.
	Children received gift packs of colored pencils and Eagle Books stickers and played with Eagle Books puppets and art activity sheets. They participated in 2 activities to assess their knowledge of Eagle Books nutrition and physical activity messages. Children drew lines on a worksheet between Mr. Eagle and activities that he would approve (playing ball) or not approve (playing video games). They sorted pictures of foods onto a Miss Rabbit plate (healthy choices) and a Mr. Coyote plate (less healthy choices).
Observational tours	The site visit team made observational tours arranged by the tribes, including local schools, Head Start programs, community colleges, tribal museums, cultural centers, casinos, tribally owned restaurants, hospitals, health departments (including an office dedicated to an Eagle Books program), tribal markets, grocery stores, economic development offices, and an Eagle Books play performance.
	We observed local use and dissemination of Eagle books, community infrastructure, and economic development. We shared dinners with tribal members, danced with elders at a weekly exercise class, listened to children reading letters to Mr. Eagle, and participated in a blessing of the tribe’s bison herd.
Team adaptability	The research team adapted to community situations. Inclement weather led to telephone interviews with participants in Alaska. We were respectful and supportive when unexpected events affected the community.
**Data analysis**	
Who conducted analysis (39)	Westat team members were assigned a set of communities for reporting and analysis based on the sites they had visited.
How analysis was conducted	The team developed verbatim transcripts for each community from audiotaped interviews and focus groups.
	These transcripts served as the primary data source for description and analysis of participant responses, observation notes, and relevant documents.
	Initially, descriptive summary reports were developed for each community. These reports included 3 sections: A brief tribal history and a description of community population, government, economy, and public services. A description of tribal health programs, schools, museums, and community organizations that had adopted the books for development of new Eagle Books–based programming or use in existing health promotion and diabetes prevention programs. A summary of community responses that included health department personnel, teachers and librarians, local college partners, school administrators, and parents and children.
Cross-site theme analysis	The community reports and original transcripts were the sources for a cross-site thematic analysis.
	Already familiar with these data and understanding that we observed in similar responses across communities, we used hand coding to develop word codes, code data, and categorize codes to discover trends and patterns.
	By combining codes and patterns, we identified broader themes, recognizing commonalities and relationships across community data.
Ensuring reliability	Because interpretation was often required, other Westat team members who had visited the same community regularly reviewed the emerging analyses, which strengthened reliability.
Triangulation and internal validity	Patterns and themes identified from multiple cases and multiple data collection methods and sources provided the opportunity to compare data and reduce errors in interpretation through triangulation.
	Alignment of these data provided evidence of internal validity and greater confidence in findings related to the appeal of Eagle Books, initial uptake and continued use, kinds of uptake and their effects, and contextual factors that influenced community-specific use of books.
	The multiple cases coupled with multiple methods of qualitative data collection (in-depth and group interviews, community observation data, and examination of program materials) enabled us to triangulate these data.
	Triangulation strengthened the findings in relation to the research questions and increased internal validity by deriving findings from multiple sources reflecting real-world community settings and populations.
**Definitions and resources**	
Participatory research and evaluation	Participatory research frameworks encompass research and program evaluation designs and methods that use systematic inquiry in direct collaboration with persons, groups, and communities that are the focus of study.
	Researchers and evaluators use methods and tools that bring participants directly into the research and evaluation process.
	Researchers, evaluators, and participants collaborate as partners to determine questions for inquiry and the means to answer them.
	Balanced, interactional relationships produce value for researchers, evaluators, and participants in knowledge gained and application in the real world.
	Tribally driven participatory research takes an active, rather than passive, stance in the research process and emphasizes the critical governmental authority of AIAN tribes.
Participatory approach formed the foundation throughout Eagle Books development and promotion	NDWP adopted participatory approaches for the production and promotion of all Eagle Books products. The program recruited Native artists to illustrate the stories and funded the art direction and production services of Westat Graphics.
	This collaboration produced the Eagle Books series, novels for adolescent readers, graphic novels, and all ancillary materials.
	As books and materials were developed, they were reviewed by members of tribal communities, including children, adolescent leaders, health educators, and tribal leaders.
	We employed a Native-owned firm to promote the books nationwide at conferences, health fairs, pow-wows, and other community gatherings.
Sustainability	Sustainability is defined as the capacity to maintain program services at a level that will provide ongoing prevention and treatment of a health problem after termination of major financial, managerial, and technical assistance from an external donor.
	Sustainability includes use of program components and activities for the achievement of desirable program and population outcomes over time.
	A main component of sustainability is the ability to maintain programming and its benefits over time (40).
Resources	NDWP’s principles of practice (Box).
	L.D.C. provided guidance on AIAN talking circles and behavioral protocols in AIAN communities.
	For Eagle Books’ promotion, L.D.C. conducted talking circles, including Eagle Books talking circles, and Eagle Books promotions in 148 reservation communities.
	C.D.F. provided guidance on diabetes education in tribal schools and curriculum uptake in communities, promoting Eagle Books during and after curriculum development of the K–12 Diabetes Education in Tribal Schools program.
	C.D.F. brought scores of Eagle Books on small planes that took her to outlying villages in Alaska. There she provided teacher training on the K–12 Diabetes Education in Tribal Schools program and supplied the village with books.
	C.W. provided guidance on use of Eagle Books in tribal nations with traditional foods programs and food sovereignty practices.
	V.Z. provided guidance on cross-cultural application of Eagle Books with African American children in Head Start programs and insights on AIAN African wisdom.
	NDWP website includes all materials developed for Eagle Books promotion and use: www.cdc.gov/diabetes/ndwp/about-us/index.html.

Abbreviations: AIAN, American Indian or Alaska Native; NDWP, Native Diabetes Wellness Program.

We knew of 3 Eagle Books evaluation studies. These included 1 of the 8 communities in the case study (41,42) and 2 programs that conducted independent evaluations, the DETS curriculum and the Jump-start on a Healthy Lifestyle program in Maryland (35,43). We reviewed the literature that described the 3 programs, comparing approaches, methods, and findings with the major themes. We listed the quantitative results that demonstrated significance in relation to Eagle Books’ use and impact. We described qualitative findings and extracted illustrative verbal descriptions from participants in the case study and other programs that supported, enhanced, and broadened our understanding of identified themes.

We conducted literature searches with Google using the terms “CDC Eagle Books program evaluation” and “CDC Eagle Books.” Our criteria included programs that had participant sample sizes large enough to determine significance and employed quantitative measures or, if qualitative, had used methods with a variety of participants, similar to the case study. We found no additional Eagle Books evaluations in the literature that met these criteria.

## Results

We identified 11 major themes that addressed Eagle Books’ popularity and why they are still in demand 16 years after launch. These themes include versatility and flexibility, cultural relevance, a relatable explanation of type 2 diabetes, colorful artwork, characters with whom children identify, relevance to diverse populations, and children as change agents (Table 2). Theme 1, versatility, includes subthemes that address sustainability: easy “as is” use; integration of books into existing programs; adaptation of the books for different genres, age groups, and diverse AIAN and other cultural groups; adjustment of books for very young readers; development of new programs for classroom, home, and Head Start; and stable, embedded use of Eagle Books across tribal and nontribal organizations and programs.

**Table 2:** Major Themes, Subthemes, and Illustrative Quotes About the Eagle Books, 2006–2022

Themes and subthemes	Quotes from the Eagle Books case study, evaluation studies, and the media
**Major theme 1: The Eagle Books’ appeal is versatility and flexibility.**	
The Eagle Books are ready to use for many purposes.	Eagle Books have not required customizing. We usually need to alter materials to make them more kid-friendly or culturally appropriate . . . but the Eagle Books are ready to use so staff can easily incorporate them into current diabetes prevention efforts. [Health Department, Southeastern Tribe]
	We count images and health activities in the books for addition and subtraction. [Elementary school teacher, Great Plains Tribe]
	The health department gives away Eagle Books to participants in our Pathways and walking program. Elders at the adult tricycle race like them to give to grandkids. This year we have worked with the tribe’s museum to put on the Eagle Books exhibit. They will be distributing the books and have asked us to integrate Eagle Books health messages into the exhibit activities. All the schools are going. [Health Department, Southeastern Tribe]
	We use *Tricky Treats* when we visit a grocery store to learn about nutrition labels and then set up our own store for shopping. And we use the books to play Fear Factor, where the kids dare each other to eat healthy foods they don’t usually eat. [Boys and Girls Club staff, Southeastern Tribe]
The Eagle Books are adjustable for early childhood education.	The children I use them with are too young. . . . There are too many words on a page. So, we look at the pictures. The 3- to 4-year-olds have an attention span of a half-inch! [Community librarian, Midwest Woodlands Tribe]
	The actual wording was a little higher level for our age group. But the pictures are so vivid and there is so much going on. Teachers can be familiar with it and then just tell the story. [Early Childhood Center’s Head Start, Alaska village]
The Eagle Books can be integrated into existing programs.	This was going to be enjoyable. Something the kids could really get into. It rippled through the community and our schools. They were excited about having copies of the Eagle Books in their classrooms. [Project DESTINY, Midwest Woodlands Tribe]
	I led training sessions across Alaska promoting the Eagle Books and DETS [Diabetes Education in Tribal Schools] program. I distributed [them] to many students who are home-schooled. I encouraged schools and after-school programs to use the books so older children are reading them to younger children. [Alaska village community educator]
	Teachers have been using *Knees Lifted High* in with “I am Moving, I am Learning.” That’s a Head Start program. And linking to the culture program about traditional living — we took children on trips to see the bison herd, then talked about traditional food and coyote food. [Head Start, Great Plains Tribe]
The Eagle Books are used to create new programs.	We introduced healthy foods, created active games, promoted social interaction, and making friends, and encouraged participation of shy students, especially those with weight problems. A key message in the books is that you are not alone. Friends help each other to stay heathy. [Fitness specialist, Great Plains Tribe]
	[This program] introduces nutritional education and physical activities in classrooms with follow-up homework for students and their families. [We created] a play based on *Through the Eyes of the Eagle*, dances, songs, and music. The evaluated program has expanded to tribes throughout [our state] and to other states. Essentially, we took the stories and layered them with activities and education. [Southern Plains Tribe Eagle Books program]
	The *Through the Eyes of the Eagle:* Illustrating Healthy Living exhibit was in collaboration with Tohono O’odham Community Action as co-curators to promote understanding of [Southwest Tribe’s] history, culture, and how they are working to prevent type 2 diabetes. [Associate Director of Education, Arizona State Museum]
Eagle Books were adapted to different genres, age groups, languages, and cultural groups.	We developed a play, *Through the Eyes of the Eagle*, that would get the kids excited and create readiness for classroom activities. We created songs and lyrics, too. [Southern Plains Tribe Eagle Books program]
	We wanted to make something like the Eagle Books that would be from the Southwest and reflect local Native cultures — more of our racial and ethnic group mix. So, we wrote a comic that included Native and Hispanic teens that were skateboarders. [Associate Director of Education, Arizona State Museum]
	There were some new outdoor games we created for older kids — 6th graders. Some we based on traditional games, but we used the Eagle Book characters in them. We called one game *Coyote with the Stinky Feet*. We were thrilled when we were riding by the school, and some kids were outside playing our games! [Fitness specialist, Great Plains Tribe]
	We created songs to help African American children understand the importance of healthy eating and physical activities: “A plate full of color, fresh from the garden. Is too much sugar good for you? Oh, no! You wanna eat fruit and vegetables? You wanna drink water, not soda? You wanna play ball? Keep it away, keep it away, diabetes, keep it away!” [Lyrics by Dionne Ray and team, Jump-start on a Healthy Lifestyle, University of Maryland Eastern Shore]
Adopters reached across multiple organizations — strengthening and creating infrastructure that support Eagle Books’ uptake.	Everybody was working like crazy for the pilot! Performing arts did the play script. The culture program helped with the dance. University partners made shakers and built the garden boxes. Kids from the after-school program, 4-H, and Boys and Girls Club played the parts. Multimedia provided background music. And later, Communications took photos for our at-home activities. [Southern Plains Tribe Eagle Books program]
	I make sure copies of Eagle Books are in the library and family resource center. The center is cozy with couches and a TV. And every year I give copies to new kindergartners, too. [School librarian, Midwest Woodlands Tribe]
	I’m a member of the wellness committee, so I worked with the clinic to develop their walking literacy trail for the primary school. And I work with Head Start and the school’s smart snack program. I’m always looking for materials relevant to Native culture and ways to introduce words from our language into the lessons. [Director, Midwest Woodlands Tribe Language and Culture Commission]
	I invited anybody interested in health, American Indians, students, food banks, public health, Native health centers, the College of Education, the Cultural Center, the College of Agriculture, and people interested in art, literacy. and nutrition. I wanted to build a partnership — an internal team — that would last. With TOCA [Tohono O’odham Community Action], we did that. [Associate Director of Education, Arizona State Museum]
**Major theme 2: The Eagle Book stories are culturally meaningful.**	
The Eagle Books fit cultural traditions of storytelling.	Storytelling is a big part of our culture. And our matriarchal structure is reflected in the books’ references to Mother Earth. These [teachings] are heavily emphasized in our cultural program. [School teacher, Southeastern Tribe]
	Our tribal members believed there was a need for diabetes prevention education for children — education that included interaction between generations and traditional storytelling. [Health educator, Southeastern Tribe]
	The books fit with the tribal cultural practice of storytelling, and they are intergenerational . . . having elders reach out to the younger members of the community. [Project DESTINY, Midwest Woodlands Tribe]
	Storytelling is an effective means for educating children because storytelling crosses individual, cultural, and educational differences more powerfully than other types of teaching methods. [V.Z.*,* Jump-start on a Healthy Lifestyle, University of Maryland, Eastern Shore]
The Eagle Books are culturally sensitive and relevant.	So, thank you, God. Finally, something that has relevance and meaning for our children to relate to. [Director, Language and Culture Commission, Midwest Woodlands Tribe]
	We are thankful for the books’ culturally sensitive presentation of type 2 diabetes that reduces children’s anxiety about getting diabetes. [Health Department, Great Plains Tribe]
	Head Start’s lessons about ancestral traditions include health and nutrition messages that we connect with the Eagle Books. They fit with the tribe’s cultural program that takes students to our fishing site to teach about traditional fishing and health benefits of traditional foods. [Head Start Education and Disabilities Coordinator, Alaska village]
The Eagle Books support parents’ traditional teachings for their children.	This series of books — it says that the eagle has come to visit. The power of our prayers, every time we use an eagle bone whistle, every time we pray with that feather, you know it goes somewhere. When people need healing, the eagle comes back. This is powerful. [Great Plains Tribe parent]
	I can see how the books interact with our own teachings. When it talks about telling stories in winter. We identify that with ourselves. Things that have meaning to us, somebody that looks like us. [Great Plains Tribe parent]
	The eagle talked about Mother Earth. Mother Earth has meaning for our family. My daughter’s friend came over and said, “Who is Mother Earth?” But in our family, it means a lot. [Southern Plains Tribe parent]
The Eagle Books are relevant in communities with different cultural elements and settings.	The one criticism I heard was that [the books] did not speak directly to Alaska Natives. No bison in Alaska — it’s caribou and moose. But most kids can make the transition. Eagles are here and fish are here, and it is relevant . . . because of the skin coloring and speech pattern. [Community educator, Alaska village]
	Even though some of the tribal elements are different from our tribe, we can relate to it. Some of the characters may be different as far as our traditional stories are concerned, but I really appreciate them. They speak to the values that are important to us. [Health Clinic Wellness Manager, Alaska village]
	In the books, the rabbit is a positive, supportive character. In our tradition, rabbits, seen as tricksters, are different. In our culture, the panther is sacred, and the eagle is negative. Despite these differences, teachers are overwhelmingly supportive of the books. [Culture Program teacher, Southeastern Tribe]
**Major theme 3: The Eagle Books explain type 2 diabetes in relatable ways.**	
The Eagle Books explain type 2 diabetes and how to prevent it in a way children can understand.	Most children have heard of diabetes because someone in the family has it. Until the Eagle Books, they didn’t really understand. It was something that just happens to them. Now they know how they can keep from getting it. [Diabetes educator, Great Plains Tribe]
	*Through Eyes of the Eagle* talks about diabetes in a way that was powerful and easy for kids to understand. It would have been hard to explain diabetes to kids without these books. [Boys and Girls Club, Southeastern Tribe]
	The books provide a friendly way to introduce the word “diabetes.” [Early Childhood Center’s Head Start, Alaska village]
	Everybody would like these books because we need to stay healthy — nothing good would happen if we aren’t healthy. All the good food comes from nature . . . but the bad food is made by some person. [3rd grade child, Southern Plains Tribe]
	Educators as well as health professionals were floored — how do you explain type 2 diabetes in a way that is not complex that children in elementary grades can understand? Eagle Books provided a way to do that. [Project DESTINY, Midwest Woodlands Tribe]
“Sometimes” and “everyday” food concepts are easy for children to remember.	My mom tried to give me a brownie, but I wanted something healthy. I knew it was a “sometimes” treat and I told her. [Great Plains Tribe child]
	My dad got this type of apple and then he got some caramel and sliced the apples, and he dipped the apples in there. Is that caramel good for us? I thought about it, and I think it is a “sometimes.” [Southern Plains Tribe child]
	A tribal employee said her son still talks about our program after two years. She made brownies and he was telling how they were a “sometimes” food and he wasn’t going to eat them! [Health educator, Southern Plains Tribe]
**Major theme 4: The Eagle Books’ colorful pictures impact messaging.**	
The Eagle Books pictures are an integral part of the health messages.	It means so much to have a beautiful book to physically hold, and you can curl up in your mother’s or father’s lap. [Southern Plains Tribe parent]
	The books made me want to eat carrots and healthy food because of the pictures. [Southwestern Tribe child]
	The children were just taken in by the pictures. [3rd grade teacher, Great Plains Tribe]
The vivid colors in the Eagle Books make health messages more memorable.	The colors made a big impression. Someone told me their daughter came home and was talking about the colors of the vegetables and she wanted to make sure they had different colors [to eat]. [Health educator, Southern Plains Tribe]
	Miss Rabbit said that type 2 diabetes can get you sick. She said you need to eat fruit and try different colors. [Southern Plains Tribe child]
	When *Plate Full of Color* came out . . . when we went to lunch, they would brag, “Look at my plate, it’s colorful.” [Middle school teacher, Midwest Woodlands Tribe]
	The thing that stuck with my kids is the colors, all the colors of the fruits and vegetables. They just liked to look at the different colors of all the healthy fruits. [Great Plains Tribe parent]
**Major theme 5: The Eagle Books’ action imagery promotes children’s interpretation and activity.**	
Children interpret and act on the Eagle Books’ messages.	The eagle was sad because he didn’t see the children playing outside, and they didn’t go play like they are supposed to . . . like they used to. [Great Plains Tribe child]
	Mr. Eagle wants me to go outside and play with dinosaurs! [Great Plains Tribe child]
	You got to play games with other children like basketball that will keep you heathy. [Great Plains Tribe child]
	My kids came home from school and said we have to move! [Southern Plains Tribe parent]
	There was one part of the story where the eagle told Rain that Dances about how our people lived a long time ago. Although it was a hard life with all the hard work, they were healthy. Now our lifestyle has changed so much, our elders are ill with diabetes. [4th grader, *USA Today,* 2006]
The Eagle Books’ action imagery promotes physical activity.	There were dancers in the books, and my kids dance, so they connected with it right off. [Great Plains Tribe parent]
	*Knees Lifted High* was good for [my daughter] because she realized she should be running around and being outside. After reading that book, she would talk about exercising and what we need to do to keep healthy. If we had music on or something, she would dance. She’d be like, “This is good for you!” [Midwest Woodlands Tribe parent]
	My kids like it. That is all they are interested in is the exercise. I’ve got a stationary bike at home and they’re like, “Dad, look at me!” And upstairs, on the mattress, they’re doing sit-ups. [Midwest Woodlands Tribe parent]
	They don’t play video games as much anymore and they [the video players] get dusty. They would rather be outside running around. [Midwest Woodlands Tribe parent]
**Major theme 6: The Eagle Books promote cultural identity.**	
Native children recognize themselves in the Eagle Books.	A father came up to me and said, “My son loves these books because the little boy has long hair and his son had long hair, which was not the norm at his school. The children physically identify with the characters.” [Health educator, Southern Plains Tribe]
	They are at an age where they notice differences between them and other kids. It helps them to identify more. Little brown kids that look like them. [Midwest Woodlands Tribe parent]
	Rain is like me. He figures things out like me and is nice to friends, like me. [5th grader, Southern Plains Tribe]
The Eagle Books portray contemporary life and traditional values.	Our teachers liked that the books show present day, modern Native children. It does not give the impression that all this happened forever ago. [Project DESTINY, Midwest Woodlands Tribe]
	Just look at the pictures. The kids are in ball caps turned backwards. It would be unrealistic for these kids to be in traditional moccasins. Our families have those and wear them, but they have their little sneakers with bright stripes, too. That is what’s real to them. [Head Start coordinator, Alaska village]
	The activities look like our kids and the kids look like us — the families relate to these stories. [Head Start coordinator, Alaska village]
	Rain that Dances is a normal kid who does what modern kids do. [Southeastern Tribe child]
The Eagle Books inspire cultural pride.	The kids feel proud when they read the books. They show pictures of kids that look like them, dress like them, play like them. [Elementary school teacher, Great Plains Tribe]
	The books are excellent. We now have books we can use in the classroom that have positive images of Native Americans — books for children! [3rd grade teacher, Great Plains Tribe]
	I thought it was really cool because there were Indian kids in the books. The kids really liked it because it was Indian kids and they said, “We’re Indian!” [Great Plains Tribe parent]
	Parents said that they felt proud to share books with their kids, that their children could relate to the characters. The eagle and coyote are favorites because animals figure prominently in our tribal stories and culture. [Great Plains Tribe parent]
**Major theme 7: The Eagle Books support health literacy.**	
The Eagle Books are used to promote literacy.	We used the Eagle Books in our family program to promote literacy and nutrition. Students and families attended a read-aloud program where the principal dressed as an eagle and read the books. They shared a healthy meal, and we gave them books to take home. [Health educator, Alaska Tribe]
	We have been using Eagle Books as part of Head Start’s family literacy initiative. We have been able to engage the parents. The parents get instructions and 4 copies of the Eagle Books every year. They are asked to read the books to their children. [Head Start teacher, Great Plains Tribe]
Parents are engaged in their family’s health literacy.	We would stop reading and I would point out things like the video games, the pop, and the “sometimes” foods. I stopped at the part about the boy who didn’t like vegetables and said, “Do you see yourself? Well, just try a little bit [to taste foods] like he did.” [Great Plains Tribe parent]
	He likes us to tell stories before he goes to bed. So, we make up stories about a wolf or a dinosaur. Sometimes we bring in the eagle or the coyote and put in something about healthy eating. Just to remind him. [Great Plains Tribe parent]
	The school has family fun night which we go to once a month. You get to eat dinner. They have raffles so kids and parents can win a raffle. They took the Big Eagle Books and put them up in the hallway, gym, and cafeteria. You got to walk through the school and read the books with the kids. We got our first set of Eagle Books in Head Start [Midwest Woodlands Tribe parent]
**Major theme 8: The Eagle Books support pre-K reading readiness.**	
Pre-K children play with the books, look at the pictures, and make up their own stories.	Kids love the pictures and know the characters. They make up their own stories about the books . . . which really helps their interest in reading. [Head Start teacher, Great Plains Tribe]
	The children play with the books so much that they tear. I’ve taped and stapled them back together many times. Then I re-order copies. [Head Start coordinator, Great Plains Tribe]
Pre-K children like the bright colors and know the roles of the characters.	The 3- to 4-year-olds are drawn to the bright colors, and they understand the role of the characters in teaching them what is healthy and unhealthy. They know the coyote cannot be trusted. He tries to get them to eat unhealthy foods. [Head Start teacher, Great Plains Tribe]
**Major theme 9: The Eagle Books support children’s sense of comfort and safety.**	
The Eagle Book help children feel better about themselves. The Eagle Books help to discuss diabetes in hopeful, not scary, ways.	The program uses Eagle Books to build resilience through activities related to traditional hunting and healthy food gathering. [Community college educator, program for troubled middle schoolers, Great Plains Tribe]
	I have a 5th-grade volleyball team. A lot of girls are overweight, and some are Native girls. They were quiet and kept to themselves. I was able to pull them out of their shells during Eagle Books lessons. [Fitness specialist, Great Plains Tribe]
	The Eagle Books are used in acanthosis nigricans screening. Health department staff noted the books’ sensitive presentation of type 2 diabetes helped reduce children’s anxiety about being screened. [Health Department, Great Plains Tribe]
	Rain and his friends support each other. I would like to have friends like that. [Great Plains Tribe child]
	The Eagle Books gave us permission to talk about diabetes in a storytelling way — simple, but accurate and hopeful — rather than talking about a terrible, scary disease. [Project DESTINY, Midwest Woodlands Tribe]
**Major theme 10: The Eagle Books appeal to diverse populations.**	
Non-Native children relate to the Eagle Books.	I brought them to read to my grandchildren We talked about the coyote being the trickster. [Non-Native community librarian noting that all children seemed to identify with the books’ characters, whether Native or not, Midwest Woodlands Tribe]
	My son was introduced to the books when he was in the child development lab at the university. I read them to his class and the children just surrounded me because they were so captivated by the artwork. [Non-Native educator, Southern Plains Tribe]
	Seeing the characters life size; they are in awe every time they see Mr. Eagle. [Health educator, referring to multi-ethnic classrooms, Southern Plains Tribe]
	Our family has read all 4 books. We are Latino, but we can relate to the books’ messages because a grandfather has diabetes. [Mother and grandmother, Tucson, Arizona]
	These children are brown like mine. [African American teacher remarking on Eagle Books’ art, Jump-start on a Healthy Lifestyle, University of Maryland, Eastern Shore]
Programs serving multiple populations use the Eagle Books.	I have never seen anything like the Eagle Books. We have never had anything this colorful to keep kids’ attention, nothing so engaging. Many of our materials for kids are simplified versions of adult materials. Just the facts, no story, no characters, no engagement. [Staff member, American Diabetes Association of Tucson]
	The exhibit was a way for us to connect the Eagle Books with our global perspective, in a way that would appeal to kids of all ages. [World of Words Library staff, College of Education, University of Arizona]
	Combining the Eagle Books series stories with music, dance, visual tools, magic tricks, and gardening was inspiring and helped Head Start teachers and caregivers at school and at home better understand the message of healthy lifestyle conveyed by the Eagle books.” [V.Z., Jump-start on a Healthy Lifestyle, University of Maryland, Eastern Shore]
**Major theme 11: The Eagle Books encourage children as change agents.**	
No one can get communities to change faster than our children.	One aspect of Native communities is all powerful — our children. No one can get Indian communities to change faster. [L.D.C., Tribal Council, Great Plains Tribe]
	Let the kids teach the elders, and they won’t know they’re learning. The kids won’t even know they’re being the teachers. [Librarian, Midwest Woodlands Tribe]
	The children are the teachers in their own innocent, honest ways. [Healthy community program staff, Southwest Tribe]
Children teach their parents about eating healthy.	My child learned from the books, then turned on me about my habits. “Mom, that’s not good!” She brought the books to me. I said, “Why are they trying to teach you when you are so little?” She said, “To be healthy!” [Great Plains Tribe parent]
	You know on Mother’s Day where it’s “I love my mom because?” Well, my youngest son wrote, “I love my mom because she gives me healthy food to eat.” [Midwest Woodlands Tribe parent]
	My kids look through the cupboard. They say, “Now this isn’t healthy, but this is healthy.” They are always asking me if something is healthy for them. [Midwest Woodlands Tribe parent]
	When I was reading it to my kids, they were getting after me about getting out and moving around. I think I got more of it than they did! [Great Plains Tribe parent]
	I was drinking my soda on my couch, and my child said, “Mr. Eagle said soda is not good for you. You will get diarrhea.” He meant “diabetes.” [Jump-start on a Healthy Lifestyle parent, University of Maryland, Eastern Shore]
Older children teach the younger children.	My daughter didn’t read it to me, she talked about it, page by page, telling me about it. My son read it to her because she is only in kindergarten. [Southern Plains Tribe parent]
	When one of the high schools was doing the high school DETS [Diabetes Education in Tribal Schools] lessons, the kids would take the Eagle Books and go teach the little kids. [Project DESTINY, Midwest Woodlands Tribe]
	[The program] worked with the high school’s youth leadership program, taking diabetes prevention messages into the pre-K through middle school. They used the Eagle Books [to describe] type 2 diabetes. [Health educator, Southern Plains Tribe]

To illustrate application of these themes, we describe 5 programs that exemplify and promote Eagle Books sustainability and operationalize their popularity.

### Whirling Thunder Eagle Books Program, Winnebago Tribe of Nebraska

In 2009, the Whirling Thunder Wellness Program collaborated with an Eagle Books champion and NDWP consultant (L.D.C.) to promote health and early literacy through the books. Whirling Thunder introduced the series to Head Start. Each family received the books and an animated video as the children were welcomed to the program (themes 7,8 [Table 2]).

Whirling Thunder developed an Eagle Books program, 4 in-class sessions for grades 1 through 6 with new outdoor games based on the characters, classroom discussion that engaged shy students at risk for type 2 diabetes, and reinforcement of messages (eg, “sometimes” and “everyday” foods) (themes 1, 4, 5, 9 [Table 2]). In 2011, the Winnebago Tribal Council passed a resolution to continue Eagle Books in their curriculum for prekindergarten through third grade (Figure 2).

A TLDC member in 2004 and Winnebago Tribal Council member (L.D.C.) developed Eagle Books Talking Circles, an adaptation of the books for educating adults about children’s health needs. This activity became part of the many she developed and presented as part of NDWP’s 2008 partnership with the Seva Foundation to fund diabetes talking circles in AIAN communities. She conducted 148 talking circles and promotional events that included booths at health fairs and pow-wows, presentations at regional and national conferences, and book distribution to families, schools, libraries, and health departments. She understands children’s power: “One aspect of Native communities is all-powerful — that’s our children. No one can get Indian communities to change faster than their children” (theme 11 [Table 2]).

Over the years, local newspapers have reported on Eagle Books events. In spring 2022, The Winnebago Times newsletter praised a skit performed by third graders based on *Plate Full of Color* and the US Department of Agriculture’s (USDA’s) My Native Plate (44).

### *Through the Eyes of the Eagle*: Illustrating Healthy Living for Children Exhibit, Arizona State Museum and Tohono O’odham Community Action

In 2008, Lisa Falk, associate director of education at the Arizona State Museum (ASM), visited the exhibit *Through the Eyes of the Eagle*: Illustrating Healthy Living, at the National Museum of the American Indian. Falk was impressed by the extraordinary artwork that addressed a serious health issue in her community (theme 4 [Table 2]). To bring the exhibit to Tucson, she built a partnership of 9 community organizations and university departments to support an expanded version of the tour (theme 1 [Table 2]). Her goal was to promote understanding of type 2 diabetes that spoke to art, history, culture, and community health (theme 10 [Table 2]). Tohono O’odham Community Action joined the partnership to represent the Tribe’s struggle with type 2 diabetes and efforts to revitalize their agricultural and athletic traditions.

Falk (ASM) and Terrol Dew Johnson (Tohono O'odham Community Action) co-curated the exhibit, which featured O’odham historical and contemporary items relating to sport and foodways spanning 13,000 years. Concurrently, ASM partnered with the Ha:San Preparatory School and faculty to develop a comic book adaptation of the Eagle Books for middle and high school students. Falk and Ryan Huna Smith (Chemehuevi/Navajo) co-wrote the comic, *It’s Up 2 You!,* set in the Southwest. The comic includes a wise tribal elder and a skateboarder who encourages her friends to eat healthy and be physically active. Students narrated a video of *It’s Up to You!* in English, O’odham, and Spanish (theme 1 [Table 2]).

Healthy Celebration Day opened the exhibit with more than 60 activities, including a 5K run, cultural dances, storytelling, food tasting, and local tribal games. Four years in development, the exhibit was on display from October 2011 to January 2012; more than 6,000 visitors attended.

When the exhibit closed, ASM added exhibit objects to its permanent collection. The 2013 edition of Sites of Conscience devoted 10 pages to the exhibit as a collaboration that addressed the critical issue of diabetes through community planning and participation. Currently, Falk delivers a lecture on the Eagle Books’ exhibit to each new class of museology students at ASM, and the museum’s website posts the comic and a diabetes quiz for download.

### Eagle Adventure Program

Eagle Adventure was the outcome of formative research conducted by staff from the Chickasaw Nation Nutrition Services Get Fresh! Program and the Department of Nutritional Sciences at Oklahoma State University to assess the health needs of local tribal families and elders from 2006 through 2008. Their findings identified type 2 diabetes as a primary concern, with elders emphasizing the need for child education that includes storytelling and intergenerational interaction (theme 2 [Table 2]).

The research team explored program products and models that would address these needs. The Eagle Books were free, available, and culturally appropriate; there were no restrictions on use; the art was spectacular; appeal would probably extend to mixed ethnicity classrooms; and the messages aligned with USDA standards (themes 2, 4, 10 [Table 2]).

The team was awarded a USDA Supplemental Nutrition Assistance Program–Education (SNAP-Ed) Wave 1 demonstration project in 2008 to develop, pilot, and evaluate an Eagle Books school-based program in grades 1 through 3. The pilot design included developing a live play, 4 classroom sessions based on each book, and take-home materials (Nestwork) that brought families and elders into the program (theme 1 [Table 2]). The classroom sessions featured nutritional messages and physical activities that included dancing, nutrition games, and a healthy harvest activity (themes 4, 5 [Table 2]).

To develop the pilot components, the team had to reach out across many departments in tribal government and locate schools willing to participate (theme 1 [Table 2]). The pilot program, named Eagle Adventure, was implemented in spring 2010, and the program was deployed in the fall.

Since its launch, the program has spread beyond Chickasaw Nation’s tribal jurisdiction, reaching more than 6,000 students throughout Oklahoma. Eagle Adventure components remain unchanged, although staff continue to create additional activities and materials. Evaluation results demonstrated significant intentions for healthy eating and physical activity choices (Table 3). In 2019, Eagle Adventure was accepted into the USDA’s SNAP-Ed toolkit, a collection of evidence-based programs. 

**Table 3:** Four Studies Representing Three Programs That Used Quantitative Measures to Evaluate the Effectiveness of Eagle Books

Program	Source	Participants	Intervention/Methods	Results
Diabetes Education in Tribal Schools (DETS) curriculum	Dodge-Francis et al (35) tested the salience of Eagle Books for teachers and students in tribal communities as part of the DETS K-4 curriculum.	385 students in grades K–4; 25 teachers in 12 states: Alabama, California, Florida, Kansas, Michigan, Minnesota, New York, North Dakota, Oregon, South Dakota, Washington, and Wyoming.	**Intervention**: classroom use of Eagle Books as part of the DETS K–4 curriculum components. **Evaluation:** postcurriculum surveys administered to students (in class; yes/no questions) and teachers (web survey) from 2007–2008.	92% of students reported that they liked the Eagle Books and said they were “fun to read.” 100% of teachers (via web survey) agreed that stories support lesson content.
Eagle Adventure^a^	Stovall-Amos et al (41) evaluated a USDA SNAP-Ed program using the Eagle Books to address food and physical activity choices, with the goal of preventing type 2 diabetes and obesity among children in tribal communities.	370 students in first and second grade in 2 schools in Oklahoma	**Intervention:** semester-long curriculum included scripted-reading play focused on Eagle Book characters; 4 in-school lessons (including Eagle Books) led by Get Fresh! health education staff; daily announcements to reinforce messaging; and Nestwork, which included health homework and the Eagle Books. **Evaluation:** paired *t* tests, based on pre–post Likert-scale surveys, determined mean differences in students’ food choices/preferences and physical activity choices, knowledge, and preferences; yes/no responses determined student’s acceptance of program components and participation in take-home activities.	Postsurvey, students had significant increases in choice of healthy food over less healthy food, vegetable preference, and choice of physical activity over sedentary behaviors. The most significant increases were seen in the physical activity measures: The mean (SE) presurvey food choice score of 6.93 (0.07) was significantly less (*P* = .002) than the postsurvey of 7.15 (0.06). The mean (SE) presurvey vegetable preference score of 6.35 (0.08) was significantly less (*P* = .001) than the postsurvey score of 6.56 (0.07). The mean (SE) presurvey physical activity choice score of 6.52 (0.07) was significantly less (*P* < .001) than the postsurvey score of 7.11 (0.06). 90.8% of students reported they saw the Eagle Play; 95.6% liked it. At home, 78.3% read or asked a caregiver to read the Eagle Books; 64.5% asked a caregiver to buy more fruits and vegetables; 62.1% did the Eagle song and dance; 69.3% played an Eagle game; 66.1% did Nestwork.
Eagle Adventure^b^	Fox et al (42) further evaluated the USDA SNAP-Ed program using the Eagle Books to address food and physical activity choices, with the goal of preventing type 2 diabetes and obesity in children in tribal communities.	494 students in grades 1–3; 113 caregivers in Oklahoma	**Intervention:** same as described by Stovall-Amos et al (41). **Evaluation**: paired *t* tests used to determine significant differences (*P* < .01) in students’ food and physical activity preferences and desirability. Postsurvey for students reporting yes/no responses to take-home activities; and Likert-scale postsurvey for caregivers reporting “more often,” “less often,” and “about the same” for children’s eating and physical activity behaviors, and yes/no responses to family participation in take-home activities.	**Student’s food preferences and desirability over less healthy foods; physical activity preference and desirability over sedentary behaviors^c^:** Mean (SE) presurvey food preference score of 6.4 (0.07) (n = 484) was significantly less (*P* < .001) than the postscore of 6.9 (0.06). The mean (SE) presurvey food desirability score of 10.0 (0.06) (n = 488) was significantly less (*P* < .001) than the postscore of 10.3 (0.6). The mean (SE) presurvey physical activity preference score of 6.2 (0.07) (n = 491) was significantly less (*P* < .001) than the postscore of 6.8 (0.06). And the mean (SE) presurvey physical activity desirability score of 8.4 (0.06) (n = 487) was significantly less (*P* < .001) than the postscore of 8.7 (0.06). **Participation in take-home activities:** 68% of students indicated that they and their families read the Eagle Books at home; 67% asked caregivers to buy fruit; 50% asked caregivers to buy vegetables; 52% did the Eagle Books song and dance; 60% played an Eagle Books game; 67% did Nestwork. **Caregivers’ observations of children’s shopping and eating behaviors:** Caregivers reported 56% of children more often helped to buy food; 71% more often asked a caregiver to buy fruit; 51% less often asked to buy candy, soda, or sweets at the store. Caregivers reported 52% of children more often eat fruit at lunch; 57% more often eat fruit for a snack; 56% more often eat a vegetable at dinner. **Caregivers’ yes/no responses to their at-home activities:** 52% made Eagle recipes; 70% did moving activities; 84% read Eagle Books with family.
Jump-start on a Healthy Lifestyle, University of Maryland Eastern Shore	Zoumenou et al (43) evaluated the effectiveness of the Jump-start on a Healthy Lifestyle curriculum, incorporating the Eagle Books to teach African American children about type 2 diabetes prevention and healthy choices.	100 students in pre-K, grades K–3, and 40 extension nutrition educators, Head Start, and elementary school teachers in Maryland, Eastern Shore	**Intervention:** after 1- or 2-day training sessions, educators implemented Jump-start on a Healthy Lifestyle, a nutrition and physical activity education curriculum, including weekly readings of the Eagle Books during 5-week summer camps. **Evaluation:** pre–post Likert-scale surveys administered to students.	**Postsurveys reported intent to choose healthier eating and physical activity:** Elementary student preference for oranges and apples increased significantly by approximately 100% (*P* < .05) in the postsurvey. Preference for fries and cookies decreased significantly by more than 75% (*P* < .05). Elementary students’ choice of video games over exercise decreased from 23% to 5% in the postsurvey. **Students increased diabetes knowledge:** Elementary students choosing “Diabetes is when you have too much sugar” increased from 43% to 72% in the postsurvey. Understanding that both exercise and eating fruit and vegetables “keeps away diabetes” increased from 15% to 60%. **Gardening activities:** 87% of elementary students increased knowledge of planting processes and origins of food. **Teachers provided pre-K observational data:** Children remembered names of characters, the stories, and Mr. Eagle messages about children trying different foods and moving their bodies.

Abbreviations: DETS, Diabetes Education in Tribal Schools; SNAP-Ed, US Department of Agriculture Supplemental Nutrition Assistance Program–Education; USDA, US Department of Agriculture.

a Chickasaw Nation Get Fresh! and partners, including Oklahoma State University, began development of Eagle Adventure in 2008.

b The partnership expanded to include additional Oklahoma tribes after reorganizing in 2017 as Oklahoma Tribal Engagement Partners.

c Food and physical activity desirability reflects the social desirability (culturally relevant and meaningful) of foods and physical activities presented in the Eagle Play.

In 2017, the Eagle Adventure team reorganized as Oklahoma Tribal Engagement Partners (OKTEP) (theme 1 [Table 2]). They added team members, and new tribal partners adopted the program. CDC remains a partner for program sustainability, providing books to support ongoing needs. Currently, 9 tribes offer Eagle Adventure.

### “Health Is Life in Balance”: DETS

Responding to TLDC guidance to develop a culturally relevant type 2 diabetes health education curriculum for AIAN children, the National Institute of Diabetes and Digestive and Kidney Diseases established cooperative agreements with 8 tribal colleges and universities and interagency agreements with IHS and CDC. DETS is a supplement for science, social science, and health education lessons in kindergarten through 12th grade designed to meet national science education standards and include AIAN traditions (theme 2 [Table 2]) (35,36).

DETS K–4 explores health and foods, healthy life in balance, diabetes as imbalance, and harvesting Mother Earth. Tribal college and university education specialists tasked with developing these lessons described the Eagle Books as having a “wow” factor. They believed that, integrated into the curriculum, the books’ beautiful imagery, character emotion, and easy-to-understand explanation of diabetes would create excitement and effective messaging in the classroom (themes 1, 3, 4, 5 [Table 2]). As expected, most children reported liking the books, drawn to the colorful illustrations, and the fun-to-read messages (Table 3) (35,36).

When DETS rolled out across Indian Country in 2008, Eagle Books had their first widespread distribution (Figure 2). The K–4 program was well-liked in schools. However, state school policies and budget cuts made it challenging to use the curriculum in some communities. Nevertheless, DETS began spreading as educators adjusted and adapted the lesson plans for informal use in schools and integration into Head Start programs (Theme 1 [Table 2]). Six of the case study communities were using DETS. In Alaska, community educators delivered DETS directly to schools as well as to families in which children were home schooled. Amazed at the popularity of DETS and Eagle Books, these educators observed a principal, dressed in a Mr. Eagle costume, reading a book to a class. Later, children participated in a DETS balance activity. The curriculum is available online from the IHS diabetes catalog.

### Jump-start on a Healthy Lifestyle, a Head Start Program

Jump-start on a Healthy Lifestyle, Head Start Program, is a University of Maryland Eastern Shore Extension program that partners with Head Start Centers and summer camps in the tri-county area of the Lower Eastern Shore of Maryland. The program promotes health, including preventing obesity, in children from low-income families. Virginie Zoumenou, University of Maryland Eastern Shore Extension, received 2 USDA grants to develop an Eagle Books–based Jump-start program (2010–2017) that served African American families (theme 10 [Table 2]). Zoumenou et al published an article describing the development and testing of components in 4 phases (43). The evaluation included students in prekindergarten through third grade in the tri-county area of the Lower Eastern Shore. Program adaptations included teachers reading Eagle Books in short excerpts for prekindergarten, shortening book length, and introducing gardening to reinforce nutritional messages (grades 1–3) (themes 1,4 [Table 2]). Zoumenou also developed songs, music, dance, and magic tricks to create classroom enthusiasm. Qualitative results showed that children remembered character names, the stories, and shared stories with their families (theme 11 [Table 2]). Quantitative results demonstrated that book messages and gardening significantly increased children’s healthy food choices, physical activity, and knowledge of diabetes (Table 3).

NDWP did not know if the popularity of the Eagle Books, designed for AIAN communities, would cross cultural groups. Zoumenou et al suggested the stories are relevant for African Americans, whose story traditions also preserve history, mores, and cultural information, consistent with *griot* practices of West African storytellers (43).

Zoumenou et al noted other commonalities (43). African Americans and AIAN people share high rates of diabetes and a long history of oppression. The consequent histories of disenfranchisement continue to result in devastating health inequities for both populations. When Mr. Eagle gives hope that diabetes can be prevented, children may also understand that history does not determine their destiny.

## Implications for Public Health

We sought to answer 2 questions: Why have the Eagle Books maintained their appeal, and what factors sustain them? Our findings suggest that Eagle Books’ appeal is due to culturally relevant storylines, relatable characters, the emotional power and beauty of the artwork, and respectful messaging of traditional health knowledge set in current times. The stories have meaning for a wider audience than in Indian Country alone, demonstrating respect, wisdom, humor, peer support, and hope. (theme 10 [Table 2]).

An important element of remembered stories is identification with story characters (45). Many children identified with the Eagle Books’ child characters because they looked like them. These and other children were equally drawn to the colorful artwork and imagery, regardless of age, race, or ethnicity. 

We had hoped to create stories that would “always be there,” guided by tribal leaders and representatives. We did not project the application of the Eagle Books’ stories for 16 years. In public health, we tend to create shorter-lived, conventional communications. Although the sustainability of public health interventions is inherently valued, sustained applications of effective messaging with measurable outcomes are not common (40,46).

**Figure 3:**
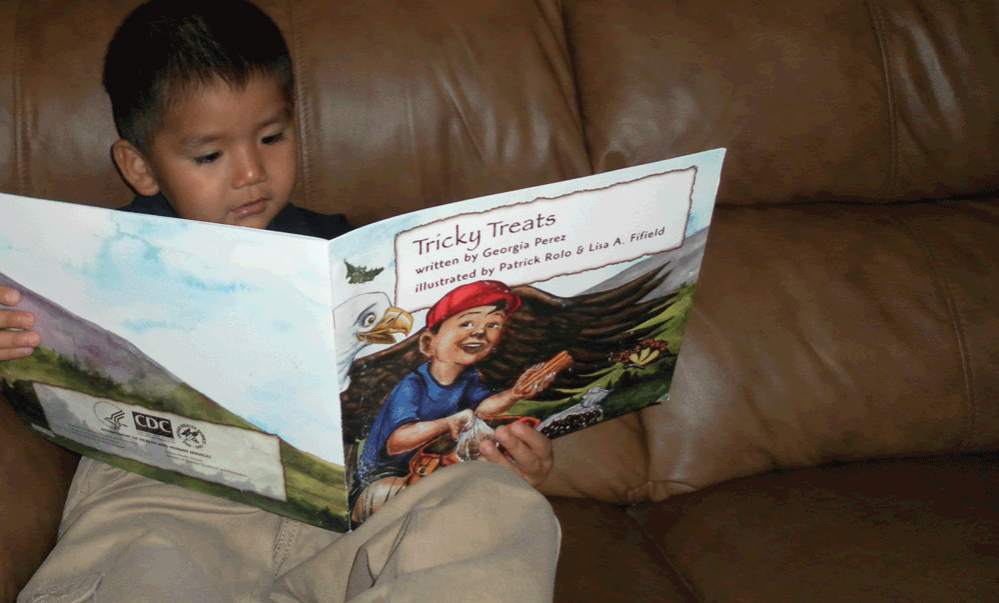
Child reading *Tricky Treats.*

The Eagle Books’ availability, including access to online versions (47), was a sustaining factor. Many children, parents, and teachers still prefer the feel of a book in hand, especially books for young children (Figure 3). The books were widely distributed — shared at events, or on request mailed to all 50 states, more than 200 tribal communities, the 6 US Affiliated Pacific Islands, Puerto Rico, and the US Virgin Islands, CDC distributed almost 6,000 sets of books annually from 2016 through 2021. The Canadian Diabetes Association tailored and printed the series. South Dakota State University Extension, through a cooperative agreement with CDC’s Racial and Ethnic Approaches in Community Health program, led talking circles and worked with native speakers to translate the series into Lakota and Dakota languages (48).

The Eagle Books inspired CDC’s National Center for Injury Prevention and Control to develop the Star Collection stories, published in 2020. *The Friendship Makers* and *Stars that Connect Us*, written and illustrated by Marisa Erven, highlight protective factors of safe, stable, and nurturing relationships (49). In 2021, OKTEP, in collaboration with CDC and the CDC Foundation, created a coloring book, *Community Protectors: Children Help Communities Stay Safe from COVID-19,* featuring original Eagle Books’ characters, illustrated by Patrick Rolo and written by James Wallace (Figure 2).

Partnerships and political infrastructures with tribal leaders and allies advocating for diabetes prevention and care are 2 of 9 domains identified as critical for program sustainability (40,46). Program evaluation, another sustainability domain, began informally when Eagle Books launched in 2006 and continues, including the recent studies (34,41–43) that confirm changes in children’s intent for healthy choices (Table 3).

In entrusting local and national partners to create paths for better health outcomes through storytelling, tribal leaders lent powerful support, grounded by traditional ecologic knowledge, to “what works,” as advised during early listening sessions. In cultures where words carry the power to shape reality, stories have the power to empower a vision of hope and strength for the future in an indirect, nonthreatening way (34).

“Stories are universal,” Zoumenou et al reminds us (43). Relatable characters enlivened by story and images can transcend cultures and bring people together. In public health, well-told stories — culturally relevant, respectfully integrating traditional knowledge with sound Western science — are a powerful tool to relay indelible messages connecting people, history, culture, hope, and health. Storyteller N. Scott Momaday deepens our understanding of stories, language, and the power of words: “Language is considered sacred and to be used in ways that count for good. Words are to be taken seriously and remembered . . . the risk of loss is constant, and language is never to be taken for granted” (50).

AIAN communities exemplify “communities of memory,” in which members share a sense of belonging, kinship, cultural identity, connectedness, and history, with understanding of the intrinsic meaning of these values for their people (51). The power of stories to create hope for the future, told and retold over generations, is time-tested. 
